# Variation in use of radiographs in chiropractic care: a cross-sectional study

**DOI:** 10.1186/s12998-025-00594-z

**Published:** 2025-08-21

**Authors:** R. K. Jensen, S. Heilmann, J. N. Thomsen, J. K. Hansen, O. Arnbjerg, C. Bell, T. S. Jensen

**Affiliations:** 1 Department of Sports Science and Clinical Biomechanics, University of Southern Denmark, Odense, Denmark; 2 Chiropractic Knowledge Hub, Odense, Denmark; 3 Private Chiropractic Practice, Næstved, Denmark; 4 Private Chiropractic Practice, Aarhus, Denmark; 5 Private Chiropractic Practice, Gentofte, Denmark; 6 Private Chiropractic Practice, Copenhagen, Denmark; 7 Medical Diagnostic Center, University Clinic for Innovative Patient Pathways, Regional Hospital Central Jutland, Silkeborg, Viborg, Denmark; 8 Department of Diagnostic Imaging, Regional Hospital Central Jutland, Silkeborg, Denmark

**Keywords:** Imaging, Radiography, Chiropractic, Healthcare

## Abstract

**Background:**

There appears to be a substantial variation in the use of radiographs in chiropractic clinics, but the reasons for this variation are not well understood. This study examined the use of radiography over a one-year period in Danish chiropractic clinics and explored its associations with clinic- and chiropractor-level characteristics.

**Methods:**

Data on the number of unique patients consulting a chiropractor and those receiving radiography between 1 January 2022 and 31 December 2022 were obtained from a Danish national registry. Information on clinics including the number of chiropractors, geographical region, multidisciplinary status, and types of other healthcare professionals employed, as well as chiropractor characteristics (age, gender, seniority, and country of education) was collected from clinic websites and a national register of Danish healthcare professionals. The proportion of patients undergoing radiography was calculated for each clinic. Associations with clinic and chiropractic characteristics were tested using chi-square or t-tests, as appropriate. Characteristics were also compared between clinics with and without in-house radiographic imaging facilities.

**Results:**

A total of 237 chiropractic clinics and 657 chiropractors were included. The mean age of chiropractors was 47 years (SD 12.8), 53% were women and 61.8% were educated in Denmark. Clinics with radiographic facilities (n = 161, 68%) tended to be larger and more likely to be multidisciplinary than clinics without (n = 76, 32%). Among clinics with radiographic facilities, the proportion of patients receiving radiography was 9.5% (95% CI 8.4–10.6%), ranging from 0 to 39%. No associations were found between radiography use in clinics with in-house radiographic facilities and clinic or chiropractic characteristics. In clinics without radiographic facilities, only 1.1% of patients were referred for radiography via chiropractic service codes, although this is likely an underestimation, as referrals to public hospitals were not captured.

**Conclusion:**

Although considerable variation in radiography use was observed across Danish chiropractic clinics with radiographic facilities, this was not explained by measured clinic or chiropractic characteristics. The true extent of radiography use in clinics without in-house facilities remains uncertain. Further research into clinical decision-making is needed to support evidence-based, transparent and consistent practice, potentially using qualitative methods to better understand the reasons behind the observed variation.

## Background

Radiography is a widely used diagnostic tool across all fields of healthcare. In 2022, more than 1,600,000 radiological examinations of the spine and upper and lower extremities were conducted in Danish public and private hospitals [1]. In chiropractic practice, radiological examinations are primarily used to assess spinal pain conditions. However, it carries certain risks, such as exposure to ionising radiation, increased healthcare costs, unnecessary investigations due to incidental findings, and poorer patient outcomes [2]. In response, there has been an increasing focus in recent years on reducing the use of imaging in spinal pain conditions, as advocated in clinical guidelines [3–5], educational literature for clinicians [6–9], and public health campaigns such as “Choosing Wisely” and national clinical care standards [10].

Despite these efforts, substantial variability in the use of radiography remains in chiropractic practice. A narrative review by Jenkins et al. (2018) reported that the proportion of chiropractic patients receiving radiographs ranged from 8 to 84% [11]. This variation includes both overuse of imaging in the relatively large group of patients with short duration of pain for which there is no indication (overall estimate: 27.7%) and underuse in the small group of patients with suspected serious pathology (overall estimate: 60.8%) according to a systematic review [12].

Over the past decade, the proportion of Danish chiropractic patients receiving radiography has declined from 15 to 8%. Nevertheless, regional data have demonstrated considerable variation [13]. For instance, in the Region of Southern Denmark in 2018, the proportion of patients receiving radiographs at their initial chiropractic visit ranged from 0% to over 65% across clinics [14]. While some of this variation may be due to legitimate differences, such as patient demographics, access to imaging facilities, prior imaging conducted elsewhere, or differing reasons for seeking care, it is unlikely that these natural variations alone account for the wide range observed. Indeed, some of the variation may be due to subjective clinical judgement and different interpretations of imaging guidelines among practitioners.

International clinical imaging guideline (2008) [15] and national Danish (2013) guidelines [16] have been published by the chiropractic profession to promote appropriate use and reduce unnecessary imaging examinations. However, these guidelines have not been updated since their publication and, currently, no valid national clinical guidelines exist in Denmark specifically for the use of radiography in musculoskeletal pain conditions. This lack of updated, official guidelines may influence the variation in utilisation rates in chiropractic practice.

Although several studies have examined chiropractic practitioners’ adherence to imaging guidelines [11, 12, 17–20] there is limited research examining how specific chiropractor or clinic characteristics relate to actual imaging utilisation [21]. Additionally, little is known about national-level variation within Denmark. Understanding these patterns could provide valuable insights into potential systematic overuse or underuse of radiography among chiropractors.

Therefore, the objectives of this study were to investigate the variation in the utilisation of radiographs over a one-year period in Danish chiropractic clinics with in-house radiographic facilities, and to explore the associations between clinic-level characteristics and imaging utilisation rates.

## Methods

This cross-sectional study adheres to the Strengthening the Reporting of Observational Studies in Epidemiology (STROBE) statement for reporting of observational studies [22].

### Setting

The healthcare system in Denmark is predominantly publicly funded, ensuring citizens have free access to hospital services and general practitioners, as well as partial reimbursement for physiotherapy and chiropractic care [23, 24]. Patients have direct access to chiropractic care, and no referral is required. They pay the reduced fee, and chiropractic clinics use the patient’s civil registration number (CPR number) and a unique clinic identification number (Clinic ID) to claim reimbursement from the Danish Regions. Reimbursements are tracked using the Clinic ID rather than an individual chiropractor ID, meaning that multiple chiropractors working at the same clinic share a single identification number. The Danish Regions record all reimbursements to chiropractic clinics operating under the national collective agreement, including those for imaging services, using specific billing codes [25]. Chiropractors are required to follow current imaging guidelines, and reimbursement for imaging referrals is made without a formal review or control system.

### Data collection and study populations

Anonymised data from 1 January to 31 December 2022, were extracted from a national registry of chiropractic service codes administrated by the Danish Regions. The dataset included the number of unique patients who consulted a chiropractor during the study period. This information was identified from billing code data covering all chiropractic visits. Also, the number of unique patients who underwent radiography in chiropractic clinics with in-house radiography facilities were identified from billing code data covering radiographic procedures performed. Data were stratified by the unique chiropractic clinic identification number (Clinic ID). Radiographic referrals from clinics without in-house imaging facilities were identified using service code 2015 (‘Primary radiography by referral from another chiropractor’), which is recorded by the receiving clinic with radiographic facilities. According to the national reimbursement agreement, clinics without imaging equipment must refer patients to an affiliated chiropractic clinic with such facilities. However, referrals from chiropractic clinics to public hospitals are not captured in the registry and could therefore not be included in the analysis.

The study population consisted of Danish chiropractic clinics operating under the collective agreement between the Danish Chiropractic Association and the Danish Regions [25]. Clinic IDs were included if they recorded any reimbursement activity in 2022. Clinic IDs that were deactivated or established during the observation period were excluded. In the registry, clinics in different geographical locations but with the same ownership may share a single Clinic ID, while a clinic with multiple owners may be registered under multiple Clinic IDs. To address this, individual chiropractic clinics were identified using multiple data sources and manually matched compile a final list of all clinics with recorded service activity.

Data on clinic and chiropractor characteristics were collected from clinic websites. Individual chiropractor data were obtained from an online register of Danish healthcare professionals maintained by the Danish Patient Safety Authority [26]. This register includes information on licensed practitioners such as age, seniority, and country of education. Two authors independently extracted data and entered them into a structured data entry form. In cases of discrepancies, the information was reviewed collaboratively, and consensus was reached through discussion.

### Variables

Imaging service codes included the following: 2014 ‘Primary radiography of own patient’ and 2015 ‘Primary radiography by referral from another chiropractor’ [25]. Information collected from clinic websites and the Authorisation Register included the presence of on-site radiographic facilities and the clinic’s geographical location (region). Additionally, data were collected on each chiropractor affiliated with the clinic, including age, gender, seniority and country of education (Denmark, UK, USA, or other countries). Further information included the total number of chiropractors employed at the clinic, as well as other healthcare professionals (e.g. physiotherapists, occupational therapists, osteopaths, medical doctors, massage therapists, craniosacral therapists).

### Statistical methods

Descriptive statistics were used to summarise chiropractor and clinic characteristics: frequency and percentage were reported for categorical variables, and means, standard deviations (SD), and ranges for continuous variables.

The overall proportion of patients who received radiography in 2022 was calculated for the entire sample, and the result was reported as the mean with a 95% confidence interval (CI). In addition, clinic-level proportions were calculated and graphically presented for clinics with radiographic facilities. For 12 clinics without radiographic facilities, 227 service codes for ‘Primary radiography of own patient’ (code 2014) and 12 service codes for ‘Primary radiography by referral from another chiropractor’ (code 2015) were registered. These entries were deemed registration errors and excluded from the analysis.

Among clinics with radiographic facilities, associations between the proportion of patients receiving radiographs and clinic-level characteristics, such as the number of chiropractic practitioners, geographical region, multidisciplinary status, and the types of healthcare professionals employed, were tested using the Wilcoxon rank-sum test or Kruskal–Wallis, as appropriate. The same statistical tests were used to explore associations with practitioner-level characteristics.

Chiropractor age was analysed both as a continues variable (mean age of all chiropractors in the clinic) and as a categorial variable (clinics with or without at least one chiropractor aged over 50). The gender distribution was categorised as clinics with only female chiropractors, only male chiropractors, or a mix of both. The proportion of female chiropractors in each clinic was also calculated. Clinics were classified according to whether all chiropractors were educated in Denmark or whether some were educated abroad. A separate analysis compared clinics with at least one US-educated chiropractor to those with none. Years since graduation was calculated as the mean number of years since graduation for all chiropractic practitioners in the clinic. Multidisciplinary status was categorised into three groups: clinics with only authorised healthcare professionals other than chiropractors (e.g. medical doctors, physiotherapists, osteopaths, occupational therapists), clinics with only unauthorised healthcare professionals (e.g. massage therapists, craniosacral therapist, reflexologist), and clinics with both authorised and unauthorised healthcare professionals.

Differences in clinic and chiropractor characteristics between clinics with and without radiographic facilities were assessed using Chi-square (X^2^) tests for categorical variables and t-tests for continuous variables, as appropriate.

All data management and analyses were performed using STATA version 17 (Stata Corp LLC, Collage Station, TX, USA).

## Results

A total of 237 chiropractic clinics were identified, of which 161 (67.9%) had on-site radiographic facilities. In total, 657 chiropractors were included, with a mean age of 47 years (SD 12.8), 53% were women, and the majority (61.8%) were educated in Denmark. Among the clinics, 77 (32.5%) were solo practices. The median number of chiropractors per clinic (including clinics with multiple locations) was 2 (IQR 1–3), with a range from 1 to 18. More than half of the clinics (n = 137, 57.8%) were classified as multidisciplinary (Table 1).

**Table 1:** Characteristics of clinics and chiropractors

	Total
*Chiropractors, n *	657
Age, mean (SD)	46.8 (12.8)
Age range	26–80
Female, n (%)	349 (53)
Country of education, n (%)	
Denmark	406 (61.8)
UK	93 (14.2)
US	138 (21.0)
Other	20 (3.0)
Years since graduation, mean (SD)	17 (12.9)
Years since graduation, range	0–50
*Clinics, n *	237
Number of chiropractors in the clinic, n (%)	
1	77 (32.5)
2–3	92 (38.8)
4–5	47 (19.8)
> 5	21 (8.9)
Multidisciplinary clinics, n (%)	137 (57.8)
Clinics with authorised professions, n (%)	30 (21.9)
Clinics with unauthorised professions, n (%)	58 (42.3)
Clinics with both authorised and unauthorised professions, n (%)	49 (35.8)
Clinic location by region, n (%)	
North Denmark Region	24 (10.1)
Central Denmark Region	46 (19.4)
Region of Southern Denmark	63 (26.6)
Region Zealand	32 (13.5)
Capital Region of Denmark	72 (30.4)

### Variation in use of radiographs between clinics

A total of 369,227 unique chiropractic patients were registered in 2022, of whom 26,831 received radiography (service code 2014 and 2015), corresponding to an overall proportion of 7.3% (95% CI 7.2–7.4%).

**Fig. 1:**
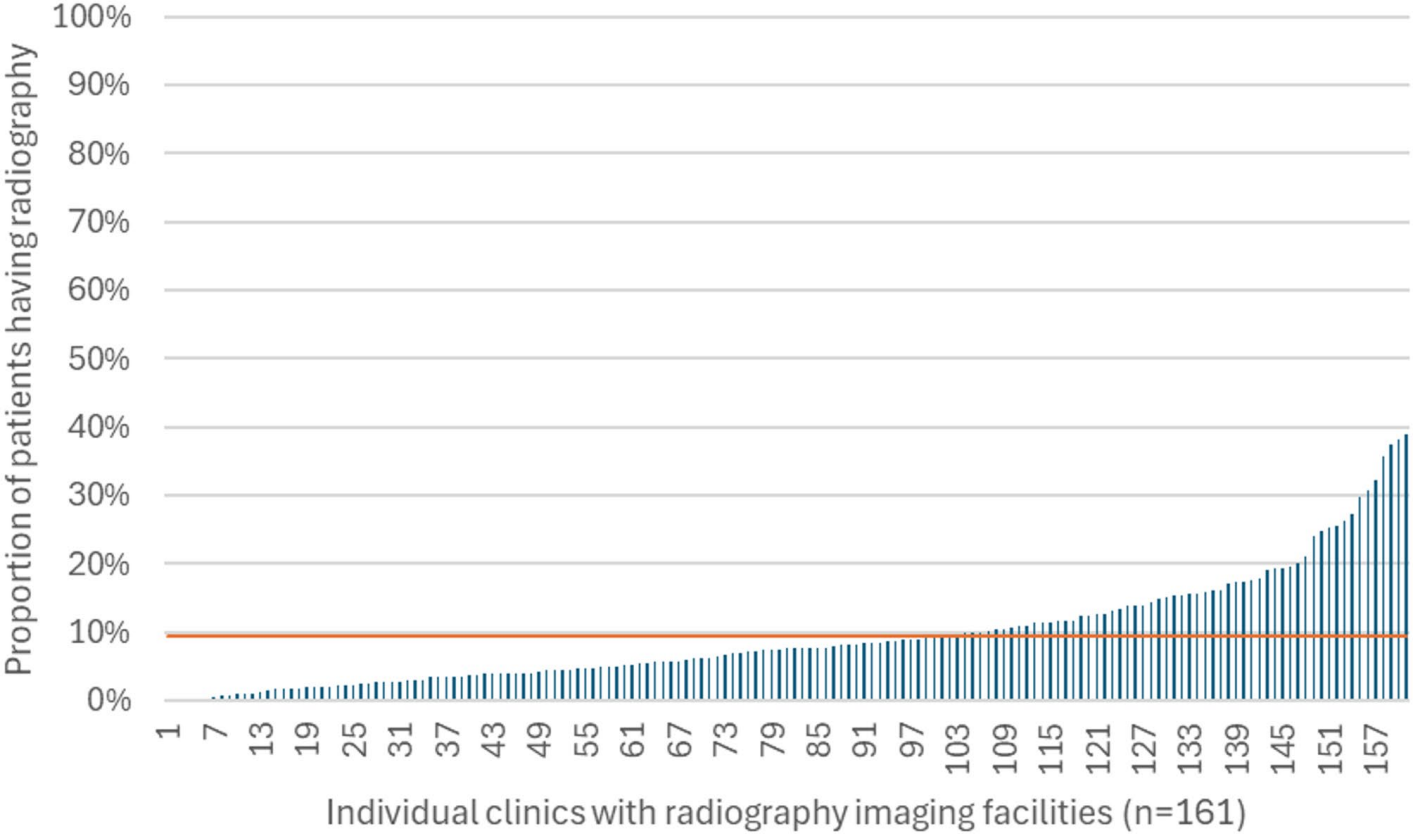
Proportion of patients receiving radiography by clinic. *Service code 2014: ‘primary radiography of own patient’. Red line illustrates mean*

Among clinics with radiographic facilities (n = 161), the proportion of patients receiving radiography under service code 2014 (‘Primary radiography of own patient’) was 9.5 (95% CI 8.4–10.6%), with clinic-level rates ranging from 0 to 39%. In the top 25% of clinics with the highest radiography use (n = 40), the mean proportion of patients receiving radiography was 21.0% (95% CI 18.9–23.0%), and these clinics accounted for nearly half (49%) of all radiographic procedures performed. Notably, two clinics with radiographic facilities did not record any radiography service codes, and 36 clinics (22.4%) registered fewer than 50 radiographs during the year. Figure 1 illustrates the variation in the proportion of patients receiving radiography across clinics with radiographic facilities.

Service code 2015 (‘Primary radiography after referral from another chiropractor’) was used for 717 patients referred from other clinics. These referrals were among 63,978 unique patients seen at the 76 clinics without radiographic facilities, resulting in an overall referral-based proportion rate of 1.1% (95% CI 1.0–1.2%).

### Associations with radiography use in clinics with radiographic facilities

Within clinics with on-site radiographic facilities, no associations were found between the proportion of patients receiving radiography and clinic-level characteristics, including the number of chiropractic practitioners in the clinic (*p* = 0.17), geographic region (*p* = 0.73), multidisciplinary status (*p* = 0.27), or the type of healthcare providers employed (*p* = 0.35). Similarly, there were also no associations between radiography use and practitioner-level characteristics such as age (*p* = 0.77), gender (*p* = 0.42), country of education (*p* = 0.66) or years since graduation (*p* = 0.64).

### Comparison of clinics with and without radiographic facilities

Clinics with radiographic facilities were more likely to be multidisciplinary (64.0%) compared to those without radiographic facilities (44.7%). Clinics without radiographic facilities were more likely to be solo practices (50.0%) compared to those with radiographic facilities (24.2%). (Table 2) Notably, only one clinic without radiographic facilities employed more than five chiropractors, whereas 20 clinics with radiographic facilities did so (Table 1).

**Table 2:** Characteristics of clinics and chiropractors by radiographic facility availability

		Clinics with radiography		Clinics without radiography
Chiropractors, n (%)	513	(78)	144	(22)
Age, mean (95% CI)		46.5 (45.6–47.5)		47.9 (46.2–49.5)
Age range		27–77		26–80
Female, n (%, 95% CI)	266	(51.8, 48.2–55.5)	83	(57.6, 50.8–64.5)
Educated in Denmark, n (%, 95% CI)	320	(62.4, 58.9–65.9)	86	(59.7, 52.9–66.5)
Years since graduation, mean (95% CI)		17.2 (16.3–18.2)		17.8 (16.1–19.4)
Clinics, n (%)	161	(67.9)	76	(32.1)
Solo practice, n (%, 95% CI)	39	(24.2, 18.6–29.8)	38	(50.0, 40.4–59.6)
Multidisciplinary clinics, n (%, 95% CI)	103	(64.0, 57.7–70.3)	34	(44.7, 35.2–54.3)
Clinic location by region, n (%, 95% CI)				
North Denmark Region	15	(9.3, 5.5–13.1)	9	(11.8, 5.6–18.1)
Central Denmark Region	29	(18.0, 13.0–23.0)	17	(22.4, 14.4–30.4)
Region of Southern Denmark	40	(24.8, 19.2–30.5)	23	(30.3, 21.4–39.1)
Region Zealand	26	(16.2, 11.3–21.0)	6	(7.9, 2.7–13.1)
Capital Region of Denmark	51	(31.7, 25.6–37.8)	21	(27.6, 19.0–36.2)

## Discussion

Among chiropractic clinics with radiographic facilities (68%), the proportion of patients receiving radiography was 9.5% (95% CI 8.4–10.6%) with a wide variation across clinics (range: 0% to 39%). No associations were found between the proportion of patients receiving radiography and clinic-level or chiropractor-level characteristics. Clinics with radiographic facilities tended to employ more chiropractors and were more likely to be multidisciplinary than clinics without radiographic facilities.

The observed variation in radiography use is less pronounced than previously reported in the literature. For example, a narrative review by Jenkins et al. [11] identified a range from 8 to 84% (nine studies) across chiropractic practices, though most included studies were conducted in healthcare systems not directly comparable to Denmark's publicly funded system. In contrast, a Danish study based on a 2002 survey found that approximately 30% of first-time chiropractic patients underwent radiography [27], while our 2022 registry-based study found a markedly lower overall proportion of 7.3% (including both clinics with and without radiographic facilities). This downward trend is consistent with previous findings showing a national decrease from 15% in 2010 to 8% in 2020 [13].

Our findings also align with broader primary care trends. A 2019 systematic review estimated that 16.3% (95% CI 12.6–21.1%) of patients presenting with low back pain received simple imaging (plain radiography or ultrasound) in primary care settings, including general practitioners, physiotherapists, and chiropractors (seven studies, n = 1,574,236) [28]. The 7.3% overall rate in our study falls below this estimate, suggestion a relatively a relatively conservative approach to imaging in Danish chiropractic care. To our knowledge, there are no national-level studies reporting rates of radiography use among other healthcare disciplines in Denmark managing musculoskeletal conditions, such as general practitioners or physiotherapists. Therefore, it is unclear whether the variation observed in chiropractic practice is also present in other parts of the Danish healthcare system.

Despite exploring a range of potential explanatory factors, we found no significant associations between radiography utilisation and clinic or chiropractor characteristics. However, it is important to acknowledge that our analysis was conducted at the clinic level. Individual-level chiropractor data (e.g., imaging practices of specific clinicians) were not available. Consequently, variables such as practitioner age, gender, country of education and years since graduation had to be aggregated to reflect the clinic as a whole. This aggregation may have masked individual-level variations and limited our ability to detect statistically significant associations.

While our findings are consistent with previous research in some respects, there are also discrepancies. For example, in a survey-based study by Jenkins [17] neither graduation year nor educational institution was associated with imaging behaviour among Australian chiropractors. Similarly, we found no associations with country of education, although our sample was more homogeneous as over half of the chiropractors were educated at the same Danish university. In contrast, the American study by Bussieres et al. [21] found that education, region, gender, and experience was significantly correlated with rates of imaging utilisation. These divergent findings likely reflect differences in healthcare system structure, geographic scale, and chiropractic education systems. Denmark's smaller size and more centralised training may contribute to a more uniform clinical culture, potentially explaining the differences in study results.

A critical limitation is the exclusion of hospital-based radiography referrals. While clinics without in-house radiographic facilities recorded a 1.1% imaging rate via referral (service code 2015), this figure is likely to underestimates the actual imaging use, as referrals to publicly funded hospitals are not captured in the registry. Therefore, the actual radiography rate in such clinics remains uncertain. This data gap limits our ability to directly compare radiographic use between clinics with and without in-house facilities and may bias our overall estimates. However, it is plausible that having in-house radiographic facilities strongly influences utilisation patterns. Jenkins [17] found that chiropractors referring to their own facilities were significantly more likely to order imaging and were less compliant with guideline recommendations.

Another important limitation of this study is the absence of data on patient clinical features, such as presenting symptoms or complexity of cases. This restricts our ability to assess whether the use of radiography was clinically indicated, and whether differences in patient populations could explain some of the observed variation in imaging rates. It is possible that clinics with radiographic facilities attract patients with more complex or acute conditions, or that patient characteristics differ systematically between clinics with and without such facilities. Future studies should incorporate clinical information to better understand whether variation in imaging reflects differences in patient case-mix or other contextual factors.

The present study is novel in its use of national registry data covering nearly all chiropractic clinics operating under a collective agreement in Denmark. According to a 2022 report, only about 6.4% of Danish chiropractic clinics are not part of this agreement [29], suggesting that our findings are largely representative of national practice patterns. However, this also raises concerns about generalisability to the small proportion of clinics not covered by the registry, which may differ in important ways.

Data on clinic characteristics were obtained from publicly available websites and the national register of licensed healthcare professionals. While these sources were supplemented by telephone follow-up in cases of uncertainty, there remains a risk of outdated or incomplete data. More accurate and real-time clinic-level information could enhance the validity of future analyses.

The findings of this study have important clinical and research implications. While some variation in radiography use may reflect legitimate differences in patient populations or clinical judgement, the magnitude of variation observed among clinics with in-house radiographic equipment raises concerns about potential over- or underuse in certain settings. Targeted efforts may be warranted to identify clinics with disproportionately high or low imaging rates and to determine whether this reflects differences in patient complexity, clinician beliefs, or practice behaviours that are not aligned with current guidelines. Understanding these drivers is essential to support the appropriate and consistent use of imaging. Strategies such as educational outreach, audit and feedback mechanisms, and the development and implementation of updated clinical guidelines may help address this issue. Future research could also incorporate qualitative methods to explore clinician beliefs and practice behaviours in greater depth.

## Conclusion

This registry-based study provides novel insights into national-level variation in the use of radiography in Danish chiropractic clinics with in-house radiographic facilities. Despite clear guideline recommendations, substantial variation in practice remains, and no associations were found with clinic or practitioner characteristics. The true extent of radiography use in clinics without radiographic facilities remains uncertain, as referrals to public hospitals were not captured in the available data. Future research should explore potential drivers of this variation, including clinician beliefs, decision-making processes, and local contextual factors. Qualitative methods could be particularly valuable for uncovering these influences and informing more consistent, evidence-based and transparent imaging practices.

## Data Availability

No datasets were generated or analysed during the current study.

## Abbreviations

CI: Confidence interval
CPR: Civil personal registration
ID: Identification
MRI: Magnetic resonance imaging
SD: Standard deviation
UK: United Kingdom
USA: United States of America
